# School screening in Coastal Karnataka

**Published:** 2019

**Authors:** Sarita Gonsalves, Srinivas Ganagi, U Vivedkanad

**Affiliations:** * Department of Ophthalmology, Father Muller Medical College, Mangalore, India; ** Department of Ophthalmology, Wayanad Institute of Medical Sciences, India; *** ASRAM, Eluru, West Godavari District, Andhra Pradesh, India

**Keywords:** refractive error, school screening, vision 2020

## Abstract

**Objective.** The objective of this study was to determine the prevalence of visual impairment and blindness among students in 13 schools in coastal Karnataka and to determine the most common causes for the same.

**Materials and Methods.** This was a prospective cross-sectional study. A total of 833 students from 13 different schools were screened as a part of the school screening programme. The examiners were an ophthalmologist and an ophthalmic assistant. For all the students, visual acuity (VA) was measured with a Snellen chart, color vision was done using an Ishiaras chart, fundus examination was done using a direct ophthalmoscope, while students with subnormal vision (VA ≤ 6/ 9) were examined using pinhole, and referred for detailed eye examination and clinical refraction.

**Results.** The age range was from 6 to 16 years comprising 390 males and 443 females. Refractive error in either eye was present in 69 of these students of which myopic astigmatism was the commonest type. This was followed by allergic conjunctivitis 15 cases, amblyopia 3, squint 2, lids and adnexal disorders in 2 students; defective color vision was seen in 8 students, all of whom were males. Spectacle usage was found to be present only in 11 students, who showed lack of awareness concerning eye care and possibly the lack of access to health care. There were no students with retinal or posterior segment disorders.

**Conclusions.** Uncorrected refractive error is the most common visual impairment found in schools students in coastal Karnataka and there is a need for the establishment of a regular school vision-screening program to detect such problems in order to prevent the burden on society due to the long life span ahead.

## Introduction

Childhood ophthalmic disorders are important as they have an important role to play in the child’s development and quality of life. According to Vision 2020, it is said that there are 1.4 million blind children in the world of whom 1 million are in Asia. Refractive errors are the second most common treatable cause of blindness in children around the world, cataract being the first [**1**,**2**]. Other causes of visual impairment include glaucoma, amblyopia, and retinal conditions. There is limited data on the prevalence, magnitude, and causes of visual impairment in schoolchildren in coastal Karnataka. Uncorrected refractive errors remain a public health problem among children in all developing countries. It can have a considerable impact on learning and academic achievement especially in Low and Middle income countries [**3**]. This article provides an overview of school eye screening and is aimed at identifying the ocular problems in the age group of 6 to 16 years in 13 schools in this region. This data can lay a foundation for the establishment of further screening strategies in schoolchildren to reduce the load of childhood blindness and visual impairment.

## Aim

The aim of the study was to determine the prevalence of blindness and visual impairment in the schoolchildren of coastal Karnataka, to determine the type of visual impairment and to assess the reasons for the lack of treatment.

## Materials and methods 

13 primary schools were identified for this study. Permission was sought from the school’s principal and a date was set for school screening. The screening team consisted of one ophthalmologist and one ophthalmic assistant who were assigned to each school to carry out the screening. The purpose was to find out the visual acuity of primary school children in Coastal Karnataka, causes of visual problems and blindness and to offer treatment to those with treatable disorders. Demographic data such as age and sex were obtained. A total of 833 students whose age ranged from 6 to 16 years were examined. Snellens visual acuity chart was used to assess the distant visual acuity and Jaegers chart for near vision assessment. Any pupil with a visual acuity of less than 6/ 6 was re assessed with a pin hole and these students were referred to the hospital for clinical refraction. Refraction was done using a streak retinoscope and all those with refractive errors were subjected to cycloplegic refraction. Color vision was determined using the Ishiaras chart. Torch light examination was done to assess the anterior segment and direct ophthalmoscopy was done for fundus evaluation. Enumeration of eligible children per household was performed between November 2015 to November 2016, in a cluster-by-cluster sequence. Family members living and eating in the same premises were deﬁned as a household. After explaining the study’s purpose and taking a written informed consent for examination and cycloplegic refraction from members of the household, demographic details such as name, age, gender, years of schooling, and name of the school were collected for each child 7 to 15 years old. A correspondence card with the scheduled date for the eye examination at a school within reachable distance within each cluster was given. Children with spectacles were requested to bring them on the day of the examination. Children who could not keep the scheduled date were given another date within the same week.

## Clinical Examination

Eye examinations were carried out on an assigned day at each school by a single screening team consisting of 1 ophthalmologist, 2 optometrists and 2 ophthalmic assistants. Visual acuity measurements at 4 m using a retro illuminated logarithm of the minimum angle of resolution chart with tumbling-E optotypes were performed by an optometrist. For children already wearing glasses, after measuring the present wearing lens power, visual acuity was measured both with and without them. Cover test and Hirshberg’s test was used to assess ocular motility and quantify degree of tropia at 0.5 and 4 meters. Cycloplegic refraction was performed with 1% cyclopentolate. Pupillary dilation of >6 mm or more with absence of light reﬂex was considered complete cycloplegia. Refraction was performed ﬁrst with a streak retinoscope (Welch-Allyn, Skaneateles, NY) and then, independently, by a second optometrist with a handheld autorefractor (Retinomax K-Plus; Nikon, Tokyo, Japan). Subjective refraction was performed on children with unaided visual acuity 20/ 40 or worse in either eye. External eye and anterior segment was evaluated using a magnifying loupe and fundus examination was performed using direct ophthalmoscope. The ophthalmologist assigned a principal cause of visual impairment for eyes with uncorrected visual acuity 20/ 40 or worse. Children with vision that improved with refraction were prescribed and provided with free spectacles. Children needing medical or surgical treatment were referred to the ophthalmology department.

## Ethical clearance

Ethical clearance from the institution’s ethical clearance was taken. The research protocol adhered to the provision of the Declaration of Helsinki for research involving human subjects.

## Data Management and Analysis

After reviewing household enumeration and clinical examination forms, data was transferred to data entry software in the computer. Prevalence rates of visual impairment and blindness using uncorrected (unaided), presenting, and best-corrected visual acuity were calculated only on children with successful cycloplegic dilatation. Normal/ near-normal visual acuity was deﬁned as acuity of > 20/ 32, visual impairment as < 20/ 40, and (legal) blindness as < 20/ 200. Myopia was deﬁned as spherical equivalent refractive error of at least -0.50 D and hyperopia as +2.00 D or more. Refractive error data are presented only for eyes with successful cycloplegic dilation. Children were considered myopic if 1 or both eyes were myopic; hyperopic if 1 or both eyes were hyperopic, so long as neither eye was myopic; and emmetropic if neither eye was myopic or hyperopic. 

Statistical analyses were performed using SPSS. Conﬁdence intervals and P values (signiﬁcant at the P < 0.05 level) were calculated with adjustment for clustering effects associated with the sampling design. Pair wise interactions between regression model variables were assessed simultaneously using a Wald F test and considered signiﬁcant at the P < 0.10 level.

## Quality Assurance

Quality assurance was monitored throughout the study by independently conducting a repeated evaluation of uncorrected visual acuity, retinoscopy, and autorefraction in children with uncorrected visual acuity of 20/ 40 or worse (either eye), after blinding the optometrist to initial finding.

A total of 647 children, 14.0% of those examined and distributed across all ages, were subjected to quality assurance evaluations. Reproducibility of visual acuity measurements was good, with unweighted Kappa statistics of 0.81 for right eyes and 0.79 for left eyes. Ninety-nine (15.3%) of the right eye measurements differed by 1 line, 7 (1.1%) differed by 2 lines, and 1 (0.2%) by 3 lines. One hundred nine (16.8%) of the left eye measurements differed by 1 line, 5 (0.8%) by 2 lines, and none by more than 2 lines.

Mean test–retest differences (the ﬁrst measurement minus the second one) for cycloplegic retinoscopy were +0.006 + 0.244 D (standard deviation) for right eyes and - 0.018 + 0.256 D for left eyes. Neither of these differences was signiﬁcantly different from zero (paired t test, P = 0.560 and P = 0.069). Reproducibility for cycloplegic autorefraction was comparable, with mean test–retest differences of + 0.003 + 0.237 D for right eyes and - 0.011 + 0.237 D for left eyes (P = 0.772 and P = 0.246).

## Results

A total number of 809 students from 13 different schools were examined, of whom 390 were males, and 443 were females. Of these, the commonest causes of eye disorders were refractive error - 69, allergic conjunctivitis 15, amblopia 3, lid and adnexal disorders 2, squint 2, defective color vision in 8, of whom all the 69 students were males with refractive errors and only 10 were already wearing the prescribed spectacles. The most common type of refractive error was myopic astigmatism. No other problems like cataract, glaucoma, traumatic eye injury, posterior segment disorders were seen. The children who were diagnosed to have refractive errors were prescribed glasses along with amblyopia therapy and asked to follow up 3 monthly to see whether there was any improvement and to check for compliance. Those students with allergic conjunctivitis were prescribed antihistamine drops. Those with hordeolum internum were prescribed topical antibiotics. Those with squint were counseled for surgery.

## Discussion

To prevent ocular morbidity in children it is important to diagnose and treat ocular diseases early as the effects can cause a burden to the society due to the long life span of children. Assessment of visual acuity in children can be difficult due to the lack of awareness among the people. However, studies conducted among school children have shown that school screening is effective identification of cases where preventable cases of blindness and low vision can be addressed at an early age [**4**,**5**]. In addition, procured data is important in planning primary eye health care services and in the determination of relevant health policies. However, it is critical to diagnose and treat ocular conditions in children early to prevent long-term adverse effects. Schools can play a significant role in screening, identifying and treating children with ocular problems [**6**]. 

In our study, we found that refractive errors were the significant cause of visual impairment accounting for ocular conditions among the age group of 6-16 years. Among the refractive errors, myopic astigmatism was found to be commonest unlike previous studies in which myopia was shown to be the commonest type of refractive error. Most of the children with uncorrected refractive error are asymptomatic, hence screening helps in the early detection and timely interventions. Allergic conjunctivitis was the second common cause in children of this age group. Amblyopia, lid and adnexal disorders and squint were also seen in the students. However, there were no children with cataract, glaucoma, trauma, or posterior segment disorders in the children screened.

This study was community based so it could predict the prevalence of ocular morbidity in children in the community to some extent. Longitudinal large population based studies involving many schools may be needed to exactly predict the prevalence of ocular morbidity among school going children. 

However, our study had the advantages that it had a good sample size, trained ophthalmic personnel to carry out the examination, it was a community-based study, and there was a standardized protocol adopted in the screening process.

In order to reduce the burden on heath care professionals, primary care workers, and schoolteachers may be trained to screen the children and suspected abnormalities can be referred to the primary health care centre in rural areas. Ophthalmologists may visit the schools at least once a year to screen and detect ocular problems. Mothers who are the primary childcare givers may be trained in antenatal classes to identify the ocular problems in their children.

## Conclusion

School eye screening visits should be at least once a year. Schoolteachers and Primary eye care workers may be trained and utilized to carry out school screening in schools, while basic eye health classes can be taught in antenatal classes to enlighten mothers who are the primary care givers. Early detection of eye conditions in children is an advantage for management. The findings from this study help to treat all avoidable cause of visual impairment and blindness as envisaged in Vision 2020 - the Right to Sight initiative.
